# Identification and In Vitro Validation of Metabolic Reprogramming-Related Hub Genes for the Diagnosis of Subarachnoid Hemorrhage

**DOI:** 10.3390/genes17101251

**Published:** 2026-10-10

**Authors:** Jin Xue, Zhaoxiang Zhang, Wuting Wei, An Zhang, Xiaoming Zhou, Qi Wu, Shujuan Chen, Jinlong Deng, Xin Zhang

**Affiliations:** Department of Neurosurgery, Jinling Hospital, Affiliated Hospital of Medical School, Nanjing University, Nanjing 210002, China; xuejin0600206@163.com (J.X.); 15531218883@163.com (Z.Z.); njwwt3060@126.com (W.W.); majoran120@163.com (A.Z.); xiaoming20111386@163.com (X.Z.); nswuqi@163.com (Q.W.); shujuancnj@outlook.com (S.C.); doctoran120@outlook.com (J.D.)

**Keywords:** subarachnoid hemorrhage, metabolic reprogramming, diagnostic model, biomarkers, in vitro validation, hub genes

## Abstract

**Background:** Metabolic reprogramming plays a vital role in subarachnoid hemorrhage (SAH). This study aims to identify biomarkers related to metabolic reprogramming in SAH. **Methods:** Transcriptome datasets (GSE36791 and GSE73378) were downloaded from the GEO database, and the metabolic reprogramming-related genes were retrieved from the Molecular Signatures Database. Candidate genes were identified by intersecting differentially expressed genes, WGCNA module genes, and metabolic genes. Hub genes were screened via CytoHubba, machine learning, and Mendelian randomization. ROC curves were generated to evaluate the diagnostic performance of the hub genes. Additionally, functional enrichment analysis, circulating immune-cell composition analysis, and drug prediction were performed. A cellular SAH model (U251 cells) and clinical samples were used for RT-qPCR validation. **Results:** Three hub genes (IMPDH2, RPL4, and HSPA1A) were identified to develop a nomogram model, which showed good diagnostic value (AUC = 0.850) in the training dataset GSE36791. Functional analyses revealed that hub genes were involved in multiple processes, including ribosome biosynthesis, purine metabolism, and immune regulation. Circulating immune-cell composition analysis showed that neutrophils were significantly enriched in SAH patients. Molecular docking suggested favorable binding affinities of mycophenolate-IMPDH2, ataluren-RPL4, and carbamazepine-HSPA1A. In clinical samples (eight SAH patients and eight controls), IMPDH2 and RPL4 were downregulated, whereas HSPA1A was upregulated. In the cellular SAH model, IMPDH2 and HSPA1A showed expression trends consistent with the clinical samples, whereas RPL4 did not show a statistically significant difference. **Conclusions:** These findings suggest that IMPDH2, RPL4, and HSPA1A may serve as potential diagnostic biomarkers for SAH and as candidate genes for further investigation.

## 1. Introduction

Subarachnoid hemorrhage (SAH) is an acute hemorrhagic cerebrovascular disease caused by the rupture of blood vessels at the base or surface of the brain, with blood flowing into the subarachnoid space [1,2]. SAH is characterized by sudden onset and rapid progression, and approximately 500,000 cases of SAH occur each year worldwide [3,4]. The clinical manifestations of SAH are diverse, and patients often experience severe headache, nausea, vomiting, and altered consciousness [5]. These manifestations overlap with those of other cerebrovascular or neurological diseases, which pose substantial challenges to the clinical diagnosis and treatment of SAH [5,6,7]. Although the diagnosis of SAH primarily relies on neuroimaging tests such as CT and CTA, these tests have limitations in certain clinical settings [8,9]. In prehospital or primary care settings where CT or CTA cannot be performed immediately, blood-based diagnostic models can aid in the early identification and triage of patients with suspected SAH [10,11]. Furthermore, blood biomarkers aid in the dynamic monitoring of delayed cerebral ischemia and cerebral vasospasm, and can assist in disease stratification and prognostic assessment to identify high-risk patients requiring close monitoring [10,12]. Consequently, the development of reliable blood-based diagnostic models for SAH holds significant clinical value—not as a replacement for imaging, but as an adjunct tool to improve early diagnosis, monitoring, and risk stratification.

Metabolic reprogramming is the process by which cells modify metabolic pathways to accommodate external environmental changes and internal physiological requirements, and it is closely related to various biological reactions [13]. Emerging evidence has shown the systemic and local metabolomic changes occurring after SAH [14,15]. In SAH patients, the levels of many metabolites, such as pyruvate, lactate, and glucose, are significantly changed, and these changes are associated with energy metabolism [16]. A study has reported that there are significant alterations in amino acid metabolism after SAH, with an overall trend of slowly elevating concentrations of all amino acid metabolites in the cerebrospinal fluid and reaching their peaks around seven days post-hemorrhage [17]. During the acute phase of SAH, lipid changes are the most significant [16]. Most major lipid categories, including sphingomyelins, free fatty acids, and phospholipids, demonstrate a significant increase immediately after the rupture of an aneurysm [18,19]. A metabolomics study displayed a shift from oxidative to glycolytic metabolism after SAH [20]. Therefore, exploring metabolic reprogramming-related genes is of great significance for the clinical diagnosis of patients with SAH. In the present study, we integrated transcriptomic datasets, metabolic reprogramming-related gene sets, differential expression analysis, and weighted gene co-expression network analysis (WGCNA) to identify SAH-associated metabolic genes. Protein–protein interaction analysis, machine-learning algorithms, and Mendelian randomization (MR) analysis were then applied to screen robust hub genes. Based on these genes, we constructed and evaluated a diagnostic model for SAH. Furthermore, we explored the potential biological functions, circulating immune-cell composition patterns, and candidate compounds associated with the hub genes. Finally, RT-qPCR validation was performed in clinical samples and in a hemin-induced cellular SAH model. This integrated analysis may provide new evidence for understanding the molecular mechanisms of SAH and identifying potential diagnostic biomarkers. The flowchart of the study design is shown in Figure 1.

## 2. Materials and Methods

### 2.1. Data Acquisition

We downloaded the datasets (GSE36791, GSE73378) from the NCBI Gene Expression Omnibus (GEO) database (http://www.ncbi.nlm.nih.gov/geo/) URL (accessed on 16 June 2025). The GSE36791 dataset (GPL10558 platform), including data from 43 SAH patients and 18 controls, was used as a training set. The GSE73378 dataset (GPL10558 platform), comprising data from 103 SAH patients and 107 controls, was used as a validation set.

Metabolic reprogramming-related gene sets were identified from the Molecular Signature Database (MSigDB) (https://www.gsea-msigdb.org/gsea/msigdb/index.jsp, accessed on 16 June 2025), and 4759 relevant genes were finally obtained (Table S1). The specific gene set names, source databases, and gene counts for each category are provided in Table S2.

### 2.2. Differentially Expressed Genes (DEGs) Analysis

The “limma” package (v 3.54.0) in R software was used to analyze DEGs between SAH samples and controls [21]. The statistically significant DEGs were determined based on |Log2FC| > 0.5 and adjusted *p* < 0.05, and the results were visualized by a volcano plot and a heatmap. A heatmap displaying the top 10 upregulated and downregulated genes was generated using the “heatmap” package. The FDR-corrected DEG list has been updated in Table S3.

### 2.3. WGCNA

The “WGCNA” package (v 1.72.5) in R software was used to construct the gene co-expression network in the GSE36791 dataset. An unsigned network was used (TOMType = “unsigned”), with a soft-thresholding power of 3 determined by pickSoftThreshold based on the scale-free topology criterion. Sample clustering (hclust, method = “complete”) was used to detect outliers; all samples were retained and none were excluded. Subsequently, a topological overlap matrix was constructed, and the dynamic tree-cutting algorithm was used to identify gene modules with minModuleSize = 200 and deepSplit = 3. Modules were merged using mergeCutHeight = 0.2. The minBranchEigennodeDissim parameter was left at its default value (equal to mergeCutHeight, i.e., 0.2), but since useBranchEigennodeDissim remained at its default FALSE, module merging was based entirely on mergeCutHeight = 0.2. Module eigengenes were calculated using moduleEigengenes, and module relationships were visualized using plotEigengeneNetworks. Other parameters included corType = “pearson”, maxPOutliers = 1, maxBlockSize = nGenes, reassignThreshold = 0, and numericLabels = TRUE. The correlation between modules and phenotypic data was calculated and visualized to identify key modules. Using ∣*cor*∣ > 0.3 and *p* < 0.05 as the general screening criterion, the turquoise module showed the strongest correlation with SAH (Cor = −0.54, *p* = 8 × 10^−6^). Lastly, genes within the key module were extracted for further analysis. To identify differentially expressed genes associated with metabolic reprogramming in SAH (MR-DEGs), the ggvenn package was used to perform an intersection analysis among DEGs, WGCNA-identified genes, and metabolic reprogramming-related gene sets.

### 2.4. Functional Enrichment Analysis and PPI Network Construction

To elucidate the biological roles of the potential MR-DEGs, GO and KEGG pathway enrichment analyses were conducted using the “clusterProfiler” package (v 4.7.1.003) in R software [22]. FDR correction was applied using the Benjamini-Hochberg method (pAdjustMethod = “fdr” for GO; pAdjustMethod = “BH” for KEGG), and terms with p.adjust < 0.05 were considered significant. Both analyses used the default clusterProfiler background: all human genes annotated in org.Hs.eg.db for GO, and all human genes annotated in KEGG hsa pathways for KEGG. GO analysis included three categories: biological processes (BP), molecular functions (MF), and cellular components (CC). KEGG analysis was utilized to explore the possible pathways involved in the pathogenesis of SAH.

To investigate the interaction relationships of 155 MR-DEGs at the protein level, a PPI network was constructed based on the STRING online website (http://string-db.org), setting an interaction score threshold >0.4 [23]. The genes without protein-protein interactions were removed, and the remaining genes were visualized by Cytoscape (v 3.9.1). To identify candidate genes, hub genes were ranked using three algorithms (MCC, Degree, and MNC) in the cytoHubba plugin. To more comprehensively include potentially important genes and reduce the risk of omission, the top 50 genes from each algorithm were selected. This step was intended as topological prioritization rather than stringent filtering. Finally, the intersections were analyzed using the “ggvenn” R package and visualized as a Venn diagram.

### 2.5. Machine Learning Analysis

To further screen key candidate genes, we developed an integrated machine-learning workflow for binary classification. We fitted 113 algorithm combinations in the training, using multiple algorithms, including SVM, RF, GBM, XGBoost, LDA, NaiveBayes, Lasso, Ridge, Enet, glmBoost, Stepglm, and plsRglm. Subsequently, we independently evaluated each model in the validation cohort. The stability and generalizability of each combination were evaluated using leave-one-out cross-validation (LOOCV). The optimal predictive model was selected according to the mean AUC [24], and the features identified by this model were defined as key candidate genes.

### 2.6. MR Analysis

SAH GWAS summary statistics were obtained from the IEU OpenGWAS database under accession number ebi-a-GCST90018923. This dataset included 473,255 individuals of European ancestry, comprising 1693 SAH cases and 471,562 controls, with 24,191,735 single-nucleotide polymorphisms (SNPs). MR analysis was conducted based on the GWAS database (https://www.ebi.ac.uk/gwas/) through the “mr” function of the “TwoSampleMR” package (v 0.5.6), combined with five algorithms: MR-Egger, weighted median, inverse variance weighted (IVW), Simple mode, and Weighted mode. SNPs strongly linking to exposure factors (*p* < 5 × 10^−5^) were identified as instrumental variables (IVs). IVs displaying significant linkage disequilibrium (LD) (clump_kb = 10, clump_r2 = 0.001) were excluded using the 1000 Genomes European reference panel (g1000_eur), and all included SNPs had F > 10. Gene expression-associated SNPs/eQTLs for candidate genes were used as exposures, and SAH GWAS summary statistics were used as the outcome. Using the IVW method, *p* < 0.05 was set as the significance threshold for the analysis results. To assess the robustness of causal inference, sensitivity analysis was performed by three tests, including the heterogeneity test, horizontal pleiotropy test, and leave-one-out test. To evaluate the directionality of the causal relationship, the MR Steiger test was performed. Instrument strength (F-statistic), multiple-testing correction (Benjamini-Hochberg FDR), and effect estimates with 95% confidence intervals are provided in Table S4. The number of instrumental SNPs for each candidate gene is provided in Table S5.

### 2.7. Construction and Assessment of Diagnostic Model

In this study, a diagnostic model based on hub genes was created by using the ‘rms’ package to predict the SAH risk. Each hub gene corresponded to a score, and the sum of the scores corresponded to the total score. The risk of SAH occurrence was predicted according to the total score, with higher scores indicating a higher risk. The performance of the nomogram model was evaluated by calculating the AUC. A calibration curve was created to display the nomogram’s predictive precision. Additionally, decision curve analysis (DCA) was performed to assess the practicality of the nomogram model.

### 2.8. GeneMANIA and GSEA Analysis

We utilized the GeneMANIA (http://www.genemania.org) database to construct a co-expression network, conduct gene function predictions, and identify genes sharing similar functions. Spearman’s correlation coefficients of the three hub genes and all other genes were calculated separately for all the samples in the dataset. Then, the genes were sorted according to the correlation coefficients from the highest to the lowest, and the corresponding lists of related genes for each hub gene were obtained. Subsequently, the “clusterProfiler” package (v 4.7.1.003) was employed for GSEA to identify the pathways associated with the hub genes. The background gene set was downloaded from the MSigDB database (https://www.gsea-msigdb.org/gsea/msigdb). Adjusted *p* < 0.05 and |NES| > 1 were set as the significance thresholds for GSEA results. The analysis results were visualized using the “enrichplot” package (v 1.18.0), and the top five positively and negatively enriched pathways for each gene were visualized.

### 2.9. Circulating Immune-Cell Composition

This study utilized CIBERSORT, MCP-counter, and ssGSEA algorithms to assess the immune cell infiltration of the samples. CIBERSORT algorithm was used to calculate the infiltration abundance of 22 immune cell subtypes and obtain the proportion of each immune cell type in each sample, with results visualized by boxplots and stacked charts. The MCP-counter algorithm calculated the distribution of eight immune cell types in SAH patients and control samples, and results were presented by a boxplot using the R package “ggpubr” (v 0.2.3). We obtained 28 immune-related cell gene sets from the TISIDB database (http://cis.hku.hk/TISIDB/) and applied the ssGSEA algorithm to calculate the infiltration enrichment score of each immune cell in each sample, with results displayed by the boxplots. Wilcoxon rank-sum test was employed to compare the differences in the expression levels of immune cells between the SAH group and the control group. Spearman’s correlation analysis was used to assess the correlation between hub gene expression levels and the relative abundance of immune cells, and the results are visualized in heatmaps.

### 2.10. Potential Drug Prediction

The DGIdb database (https://www.dgidb.org/) was utilized to identify potential therapeutic drugs for SAH, and a network diagram of targeted drugs and hub genes was created using Cytoscape (v 3.9.1). To determine the binding affinity between the predictive drugs and the hub genes, molecular docking was performed using the CB-Dock2 website (https://cadd.labshare.cn/cb-dock2/php/index.php). Firstly, the 3D structures of the hub gene proteins were downloaded from the PDB database (https://www.rcsb.org/), and the 3D structures of the drugs were obtained from the PubChem database (https://pubchem.ncbi.nlm.nih.gov/). The protein structures and chains used for docking are seen in Table S6.

### 2.11. Cellular SAH Model

To establish an in vitro model simulating SAH, U251 human glioma cells were stimulated with hemin. The U251 (CL-0237, STR-authenticated) cells were purchased from Wuhan Procell Life Science Technology Co., Ltd. (Wuhan, China) and were cultured in DMEM containing 10% fetal bovine serum under conditions of 37 °C and 5% CO_2_. Although U251 cells cannot fully recapitulate the cellular complexity of SAH, hemin stimulation in these cells has been used to mimic hemorrhage-associated oxidative and inflammatory injury. Therefore, this model was used as a preliminary in vitro system to validate hub-gene expression trends. U251 cells were cultured in high-glucose DMEM supplemented with 10% fetal bovine serum and 1% penicillin-streptomycin. After cell adherence, the control group (CON) was treated with fresh complete medium, while the model group (MOD) was incubated with fresh complete medium containing hemin (30 μmol/L). Following 24 h of stimulation, cells were harvested for subsequent experiments. Three biological replicates were set for each group. The success of model establishment was verified by cell counting kit-8 (CCK-8) assay, measurement of reactive oxygen species (ROS) and malondialdehyde (MDA) levels, as well as detection of proinflammatory cytokines including IL-1β, IL-6, and TNF-α. U251 cells were used as a simplified in vitro system to mimic hemin-induced oxidative and inflammatory injury, not as a full SAH model.

### 2.12. Reverse Transcription-Quantitative Polymerase Chain Reaction (RT-qPCR)

Blood samples (8 SAH and 8 normal controls) were obtained from the Affiliated Jinling Hospital, Medical School of Nanjing University, Nanjing, China. Ethical approval was granted by the Institutional Ethics Review Board of Jinling Hospital (2025DZKY-045-01), and all procedures were conducted in accordance with relevant guidelines. The clinical characteristics of participants are in Table S7. RT-qPCR was used to quantify the expression of IMPDH2, RPL4, and HSPA1A in SAH and control samples, and to quantify the expression of IL-1β, IL-6, TNF-α, IMPDH2, RPL4, and HSPA1A in the SAH model group and control group.

In brief, total RNA was extracted using an RNA extraction kit (Goonie, Guangzhou, China), and reverse transcribed into cDNA utilizing a first-strand cDNA synthesis kit (Goonie). RT-qPCR analysis was performed using the SYBR Green method. The relative mRNA expression levels were calculated using the 2^−ΔΔCt^ method, and β-actin was used as an endogenous control. The primer sequences used in this study are shown in Table S8. This study was approved by the Institutional Review Board of Jinling Hospital (Approval No. 2025DZKY-045-01). Each group included three independent biological replicates (*n* = 3), and each sample was measured in three technical replicates to assess assay stability. All human blood samples were obtained from the Department of Neurosurgery, Jinling Hospital, between July 2025 and September 2025, with written informed consent from all participants.

### 2.13. CCK-8 Assay

U251 cells were digested with trypsin to prepare a single-cell suspension. U251 cells were seeded in a 96-well plate (5000 cells/well) with 100 µL of the cell suspension and cultured in an incubator. After 24 h or 48 h of incubation, 10 µL of CCK-8 solution (100–120, Xintianweng, Hefei, China) was added to each well, followed by incubation in the dark for 30 min. The absorbance (OD value) at 450 nm was measured using a microplate reader (Thermo Fisher Scientific, Waltham, MA, USA). The data were analyzed using independent-samples *t*-tests for between-group comparisons, and data are expressed as mean ± SD. Each group included three independent biological replicates (*n* = 3), and each sample was measured in three technical replicates to assess assay stability.

### 2.14. ROS Measurement

U251 cells were digested with trypsin and centrifuged at 1000 rpm for 5 min. After discarding the supernatant, cells were resuspended in pre-warmed PBS, and the density was adjusted to 1 × 10^6^ cells/mL. Under light-protected conditions, the DCFH-DA probe (S0033, Beyotime, Shanghai, China) was diluted 1:1000 with PBS to a final concentration of 10 µmol/L. Then, the probe solution was added to the cell suspension and incubated at 37 °C for 20 min. After the incubation, cells were washed three times with PBS. The fluorescence intensity at an excitation wavelength of 488 nm and an emission wavelength of 525 nm was detected using a flow cytometer. The data were analyzed using independent-samples *t*-tests for between-group comparisons, and data are expressed as mean ± SD. Each group included three independent biological replicates (*n* = 3), and each sample was measured in three technical replicates to assess assay stability.

### 2.15. MDA Detection

U251 cells were collected, washed twice with PBS, lysed fully with lysis buffer, and frozen in liquid nitrogen and thawed repeatedly for 5 cycles. After centrifugation at 8000× *g* for 10 min at 4 °C, the supernatant was collected. An aliquot of the supernatant was used for BCA quantification, and the rest was mixed with TBA reagent. After heating in a boiling water bath for 60–90 min, the mixture was cooled to room temperature and centrifuged at 10,000× *g* for 10 min. Then, 200 µL of the supernatant was transferred to a 96-well plate. The absorbance at 532 nm and 600 nm was measured using a microplate reader, and intracellular MDA content (BC6415, Solarbio, Beijing, China) was calculated according to the manufacturer’s instructions. The data were analyzed using independent-samples *t*-tests for between-group comparisons, and data are expressed as mean ± SD. Each group included three independent biological replicates (*n* = 3), and each sample was measured in three technical replicates to assess assay stability.

### 2.16. Statistical Analysis

Bioinformatics analyses in this study were performed using R software (v 4.2.2). Wilcoxon rank-sum test was used to compare the expression levels of HSPA1A, RPL4, and IMPDH2 between the SAH group and the control group. The diagnostic performance of HSPA1A, RPL4, and IMPDH2 was separately evaluated by calculating the AUC. Spearman’s correlation analysis was performed to assess the relationship among hub genes. Statistical significance was defined as adjusted *p* < 0.05. CCK-8, ROS, MDA, and cytokine assays were analyzed using independent-samples *t*-tests for between-group comparisons, and data are expressed as mean ± SD. All statistical analyses were performed using GraphPad Prism 9.0. *p* < 0.05 was considered statistically significant. Each group included three independent biological replicates (*n* = 3), and each sample was measured in three technical replicates to assess assay stability.

## 3. Results

### 3.1. Identification of DEGs and SAH-Related Module Using WGCNA

A total of 775 DEGs remained after FDR correction (FDR < 0.05); the original *p* < 0.05 analysis identified 819 DEGs, including 375 upregulated genes and 444 downregulated genes (Figure 2A), and a heatmap displayed the top 10 upregulated and downregulated genes (Figure 2B). WGCNA was utilized to identify interrelated gene clusters or modules that were closely associated with SAH. The optimal soft threshold power, β = 3, was selected based on scale independence (r^2^ = 0.85) and mean connectivity (Figure 2C). Dynamic tree cutting produced 13 gene co-expression modules related to SAH when the minimum gene number for each module was set at 200, and deepSplit was set to 3 (Figure 2D). Using ∣*cor*∣ > 0.3 and *p* < 0.05 as the general screening criterion, the turquoise module showed the strongest correlation with SAH (Cor = −0.54, *p* = 8 × 10^−6^). The blue module also showed a significant correlation (Cor = 0.45, *p* = 3 × 10^−4^), but was weaker than the turquoise module; therefore, the turquoise module was selected as the primary module for downstream analysis (Figure 2E). Consequently, 5472 genes within the turquoise module were chosen for further study. The Gene Significance (GS) versus Module Membership (MM) for the turquoise module is showed in Figure S1. All genes in each module together with their module assignment, MM within their respective module, and GS for SAH status are showed in Table S9.

### 3.2. Enrichment and Interaction Analysis of SAH-Related Genes

A total of 155 MR-DEGs were obtained by intersecting 819 DEGs, 5472 turquoise module genes, and 4759 metabolic reprogramming-related genes (Figure 3A). GO analysis revealed that these genes may play significant roles in biological processes such as ribosome biogenesis, purine nucleotide metabolism, and lymphocyte proliferation (Figure 3B). KEGG analysis revealed that these genes may be involved in the HIF signaling pathway, NOD-like receptor signaling pathway, and lipid and atherosclerosis (Figure 3C). To investigate interactions among these proteins, a PPI network was established, comprising 137 nodes and 1085 edges (Figure 3D). To more comprehensively include potentially important genes and reduce the risk of omission, Figure 3E displays the top 50 significant genes selected by MCC, Degree, and MNC algorithms.

### 3.3. Identification and Clinical Validation of Three Hub Genes

After intersecting the genes identified by the three CytoHubba algorithms, 48 candidate genes were obtained for further analysis (Figure 4A). This step was intended for topological prioritization rather than stringent filtering, and the high overlap among the three algorithms (48 out of 50 genes in common) indicates that the topologically important genes were highly consistent. These 48 genes were then used as input for subsequent machine-learning and MR analyses to reduce dimensionality and computational burden while preserving the most connected candidates. To further identify hub genes, 113 machine learning algorithm combinations were evaluated, and Stepglm[backward] + RF showed the best performance, with the highest average AUC of 0.854 (Figure 4B). Subsequently, MR analysis identified six significant genes using the IVW method, including ITGAM, BCL2, RPL4, IMPDH2, RPS23, and HSPA1A (Figure 4C). The reliability of these results was supported by the exposure–outcome association plots (Figure S2), the estimated effects of instrumental variables on the outcome (Figure S3), and sensitivity analyses, including the horizontal pleiotropy test (Table S10), leave-one-out analysis (Figure S4), and Steiger directionality test (Table S11). However, the heterogeneity test revealed significant heterogeneity for BCL2 (Table S12). In addition, RPS23 and ITGAM were not retained because their transcriptomic expression patterns were not consistent across diagnostic and causal-inference analyses (Figure 4D).

Correlation analysis showed that IMPDH2 was positively correlated with RPL4, whereas HSPA1A was negatively correlated with both IMPDH2 and RPL4 (Figure S5). RT-qPCR validation further showed that IMPDH2 and RPL4 were downregulated, whereas HSPA1A was upregulated in SAH clinical samples compared with controls (Figure 4E), which was consistent with the bioinformatics results. It should be noted that this clinical validation was based on a small cohort of eight SAH patients and eight healthy controls, and therefore these findings should be interpreted as preliminary.

### 3.4. Construction and Evaluation of the Diagnostic Model

As shown in Figure 5A, IMPDH2, RPL4, and HSPA1A all exhibited good diagnostic performance for SAH, with AUC values greater than 0.7. To estimate the risk of SAH, we constructed a nomogram model based on these genes (Figure 5B). To validate the model’s accuracy, clinical utility, and discrimination, we conducted calibration, DCA, and ROC analyses. The calibration curve showed good agreement between the predicted and observed probabilities (Figure 5C), and DCA indicated the potential clinical utility of the model (Figure 5D). In addition, the nomogram model had good diagnostic performance, with an AUC of 0.85 (95% CI: 0.726–0.974). The optimal cutoff by the Youden index was 0.151, at which sensitivity was 0.778 and specificity was 0.930. Calibration was performed with 1000 bootstrap repetitions and showed good agreement between predicted and observed probabilities (Figure 5E).

### 3.5. Exploring the Potential Biological Functions of Hub Genes

GeneMANIA analysis suggested that these genes were mainly involved in purine nucleoside monophosphate metabolism, nucleoside monophosphate metabolism, and the purine nucleotide biosynthesis process (Figure 6A).

To further elucidate the biological processes and potential molecular pathways associated with the hub genes, we performed GSEA for IMPDH2, RPL4, and HSPA1A. IMPDH2 and RPL4 were enriched in pathways such as cytokine-cytokine receptor interactions and gap junctions, whereas HSPA1A was enriched in NOD-like receptor, Toll-like receptor, and leukocyte transendothelial migration pathways (Figure 6B,D,F). KEGG analysis further revealed that IMPDH2 and RPL4 associated with pathways such as cytokine-cytokine receptor interactions and gap junctions, while HSPA1A is associated with signaling pathways such as NOD-like receptors, Toll-like receptors, and leukocyte transendothelial migration (Figure 6C,E,G).

### 3.6. Circulating Immune-Cell Composition Analysis and Its Correlation with Hub Genes

To evaluate the relationship between SAH and immune cells, we performed circulating immune-cell composition analysis using multiple algorithms. The box plot and the stacked bar chart respectively illustrate the distribution range and relative proportion of various immune cells in SAH (Figure 7A,B). Compared with the control group, the proportions of M0 macrophages, neutrophils, and gamma delta T cells in SAH patients were significantly increased, while CD8+ T cells and NK cells were significantly decreased (Figure 7C). To further assess the robustness of the circulating immune-cell composition patterns, MCP-counter and ssGSEA analyses were performed. These analyses supported the CIBERSORT results to some extent, particularly with respect to increased neutrophil infiltration and reduced CD8+ T cell/NK cell-related enrichment in SAH samples (Figure 7E,G).

We then examined the correlations between the hub genes and immune cells. Significant associations were observed in the CIBERSORT analysis (Figure 7D), and similar trends were confirmed by MCP-counter and ssGSEA (Figure 7F,H). Notably, HSPA1A was positively correlated with neutrophils and CD8+ T cells, whereas IMPDH2 and RPL4 were negatively correlated with these immune cells.

### 3.7. Prediction and Molecular Docking of Candidate Compounds

To explore compounds potentially interacting with the hub genes, drug–gene interaction prediction was performed using the DGIdb database. A total of 10 drugs targeting IMPDH2, 5 targeting RPL4, and 4 targeting HSPA1A were identified. Among them, mycophenolate, ataluren, and carbamazepine were selected as potential approved drugs targeting IMPDH2, RPL4, and HSPA1A, respectively (Figure 8A–C). Molecular docking further supported the interactions between these drugs and their corresponding targets. Carbamazepine, ataluren, and mycophenolate showed favorable binding affinities for HSPA1A, RPL4, and IMPDH2, with docking scores of −9.4, −7.4, and −7.7 kcal/mol, respectively (Table S6). Specifically, carbamazepine formed one hydrogen bond with HSPA1A at THR-14 (Figure 8D), ataluren formed two hydrogen bonds with RPL4 at TYR-122 and ARG-33 (Figure 8E), and mycophenolate formed nine hydrogen bonds with IMPDH2, involving GLY-326, SER-327, ASP-364, ILE-367, and SER-388 (Figure 8F).

### 3.8. Validation of Hub Gene Expression in the Cellular SAH Model

To further validate the expression patterns of the hub genes in vitro, a cellular (U251) SAH model was established. Compared with the control group, the SAH model showed significantly reduced cell viability (Figure 9A), accompanied by increased ROS and MDA levels (Figure 9B,C), as well as elevated expression of IL-1β, IL-6, and TNF-α (Figure 9D–F). These results indicated that the cellular SAH model was successfully established. We then examined the expression levels of IMPDH2, RPL4, and HSPA1A in this model. IMPDH2 expression was decreased, whereas HSPA1A expression was increased in the SAH group. In contrast, RPL4 showed not significant difference between the two groups (Figure 9G–I). These findings showed that IMPDH2 and HSPA1A expression changes were consistent between the cellular model and clinical samples, whereas RPL4 was not significantly altered in vitro, indicating that the in vitro validation of the three hub genes was only partially consistent.

## 4. Discussion

SAH is a life-threatening cerebrovascular emergency characterized by acute bleeding into the subarachnoid space and subsequent secondary brain injury [25,26]. Increasing evidence indicates that metabolic reprogramming is involved in the pathological progression of SAH, including energy metabolism disturbance, oxidative stress, lipid metabolic alterations, and inflammatory activation [27]. Although SAH diagnosis primarily relies on imaging such as CT/CTA, blood biomarkers could assist in early identification and triage in prehospital or primary care settings where immediate imaging is unavailable. They may also facilitate dynamic monitoring of delayed cerebral ischemia and cerebral vasospasm, as well as disease stratification and prognosis assessment. These biomarkers should not be considered a replacement for imaging, but rather as complementary tools when imaging results are equivocal or repeated examinations are needed. Therefore, identifying metabolic reprogramming-related biomarkers may improve our understanding of SAH pathogenesis and provide potential tools for early diagnosis. In this study, we integrated bioinformatics analysis, machine-learning screening, MR analysis, circulating immune-cell composition analysis, molecular docking, and experimental validation to identify three hub genes, IMPDH2, RPL4, and HSPA1A, associated with SAH.

This study identified 155 candidate genes by integrating DEGs, WGCNA modules, and a metabolic reprogramming gene set. GO and KEGG enrichment analyses indicated that these candidate genes may be involved in ribosome biogenesis, purine metabolism, and cellular signal transduction, which have been reported as important biological processes of SAH [28]. Based on the PPI network, the CytoHubba algorithm, and machine learning, 12 key candidate genes were further selected. Finally, MR analysis was used to verify the potential causal association, and 3 hub genes (IMPDH2, RPL4, and HSPA1A) were identified. In the clinical samples, HSPA1A was upregulated, whereas IMPDH2 and RPL4 were downregulated. However, in the cellular SAH model, only HSPA1A and IMPDH2 showed consistent expression changes, while RPL4 was not significantly altered. The novelty of this study is threefold. First, it is the first study to integrate metabolic reprogramming-related genes with SAH diagnosis. Second, it integrates a multi-level analytical framework combining DEGs, WGCNA, machine learning, MR, immunological analysis, and experimental validation. Third, it provides preliminary validation using clinical samples and cellular models, and develops a diagnostic nomogram based on three hub genes.

Accumulating evidence supports the clinical utility of blood-based markers in SAH. Blood markers of cerebral vasospasm after aneurysmal SAH, highlighting that hyponatremia, hyperglycemia, leukocytosis, and elevated CRP and CSF IL-6 levels are associated with cerebral vasospasm [29]. These findings suggest that a biomarker panel may facilitate predictive modeling and early risk stratification. However, most of these markers are conventional laboratory parameters reflecting general inflammatory or metabolic states rather than specific molecular mechanisms. In contrast, the present study identified metabolic reprogramming-related hub genes (IMPDH2, RPL4, and HSPA1A) as more mechanistically oriented candidate biomarkers for SAH. Compared with conventional serum markers, these genes provide insights into the metabolic and immune dysregulation underlying SAH pathophysiology, thereby complementing existing blood-based biomarker approaches.

IMPDH2 protein could assemble into a specialized protein polymer fiber, and this structure was a natural biological phenomenon related to purine metabolism, functioning as a fine-tuning protein to adapt to changes in intracellular and extracellular environments [30]. A study reported that IMPDH2 fibers could protect against neurodegenerative disorders caused by AMPD2 deficiency [31]. Also, IMPDH2 was found to be linked to migraine with aura and interacts with existing drugs, indicating its potential as a candidate target for further investigation [32]. RPL4 encodes a ribosomal protein that is a component of the 60S subunit and has been reported to affect the development of tumors or diseases by involving ribosomal protein biogenesis [33,34]. The expression level of RPL4 was downregulated in MPTP-induced stomach mucosal injury and played significant signaling and core roles in immune function [35]. Notably, RPL4 was downregulated in clinical SAH samples but unchanged in the U251 model. This discrepancy may reflect the single-cell-type nature of the U251 model, differences in hemin stimulation timing and intensity, and the fact that RPL4 was identified partly through transcriptomic and MR analyses. Its validation should therefore be considered partial and model-dependent, requiring further multicellular or in vivo confirmation. HSPA1A, a member of the heat shock 70 kDa protein (HSP70) family, is associated with oxidative stress and exhibit high expression in ischemic stroke specimens and in cerebral ischemia-reperfusion injury tissues [36,37]. Furthermore, GSEA revealed that IMPDH2 and RPL4 were enriched in the cytokine-cytokine receptor interaction and Gap junction pathways, whereas HSPA1A was enriched in the NOD-like receptor signaling and Toll-like receptor signaling pathways. The association between IMPDH2 and gap junction-related pathways suggests that IMPDH2 may participate in intercellular communication and inflammatory signaling during SAH. However, whether the downregulation of IMPDH2 contributes to or reflects SAH-related pathological changes requires further experimental validation [38,39,40]. The cytokine-cytokine receptor interaction pathway regulates immune responses, and RPL4, a ribosomal protein, has been implicated in inflammatory processes, which may in turn affect SAH outcomes [41,42]. Previous studies have reported that HSPA1A interacts with the Toll-like receptor signaling pathway, thereby activating inflammatory responses and promoting the release of pro-inflammatory factors, which may exacerbate SAH-induced brain injury [25,43].

Evidence has shown that immune disorders are implicated in the pathogenesis of SAH [44]. In this study, immune infiltration analysis revealed a higher enrichment of neutrophils in the SAH group than in the control group. This result was consistent with findings from the previous studies, which indicated that neutrophils were a risk factor for SAH and tended to increase in the peripheral blood of patients with SAH [45,46]. The hub genes, IMPDH2, RPL4 and HSPA1A, were correlated with various immune cells, suggesting that they may play a crucial role in the inflammatory response. For instance, the stress-induced heat shock protein (HSPA1A) can promote neutrophil chemotaxis, further aggravating inflammation and leading to a poor prognosis in SAH [47,48].

Drug–gene interaction prediction and molecular docking suggested that mycophenolate, ataluren, and carbamazepine may interact with IMPDH2, RPL4, and HSPA1A, respectively. Mycophenolate has been approved to prevent solid-organ and hematopoietic stem cell transplant rejection, and is widely used as an immunosuppressant for autoimmune diseases, such as systemic lupus erythematosus, lupus nephritis, and pemphigus vulgaris [49,50]. Ataluren has been used as a treatment for nonsense mutation Duchenne muscular dystrophy [51]. Carbamazepine was a common drug used for epilepsy and trigeminal neuralgia, which are neurological disorders [52,53]. However, these results should be interpreted with caution. Molecular docking only predicts potential binding affinity and does not demonstrate pharmacological efficacy or clinical benefit in SAH. Further in vitro and in vivo experiments are required to determine whether these compounds can modulate the corresponding targets and influence SAH-related pathological processes.

This study has several limitations. First, although two public datasets were used, the sample size remained limited, and potential batch effects between datasets may have influenced the results. Second, the diagnostic model requires further validation in larger, independent, multicenter cohorts. More importantly, prospective evaluation in real-world clinical settings is warranted to confirm the clinical utility and generalizability of the model before it can be translated into routine practice. Inaddition, the cross-validation results of model within the training set serve as the primary evidence for model performance. Third, MR analysis provides supportive genetic evidence but cannot fully eliminate the effects of weak instruments, horizontal pleiotropy, population stratification, or tissue-specific regulatory mechanisms. Fourth, the clinical RT-qPCR validation included only 8 SAH patients and 8 healthy controls. This small validation sample limits the statistical power and generalizability of the findings, and therefore the clinical results should be interpreted as preliminary. In addition, the expression of RPL4 was not significantly changed in the U251 model despite its downregulation in clinical samples, indicating that the experimental validation of the three hub genes was not uniformly consistent. This discrepancy highlights the limitations of a single-cell-type in vitro model and suggests that the role of RPL4 in SAH requires validation in more physiologically relevant systems. Fifth, the hemin-induced U251 cell model only partially mimics hemorrhage-associated oxidative and inflammatory injury and cannot fully reproduce the complex multicellular microenvironment of SAH. Finally, the drug prediction and molecular docking analyses were computational and require experimental validation before any therapeutic implications can be drawn.

## 5. Conclusions

In conclusion, this study identified three metabolic reprogramming-related hub genes, IMPDH2, RPL4, and HSPA1A, associated with SAH. A nomogram model based on these genes demonstrated potential diagnostic value in distinguishing SAH patients from controls. Functional enrichment and immune infiltration analyses suggested that these hub genes may participate in metabolic regulation, inflammatory activation, and immune responses during SAH. Clinical and in vitro validation further supported the dysregulation of these genes, although additional studies in larger cohorts and more representative experimental models are required. These findings provide candidate biomarkers and mechanistic clues for future SAH research.

## Figures and Tables

**Figure 1:**
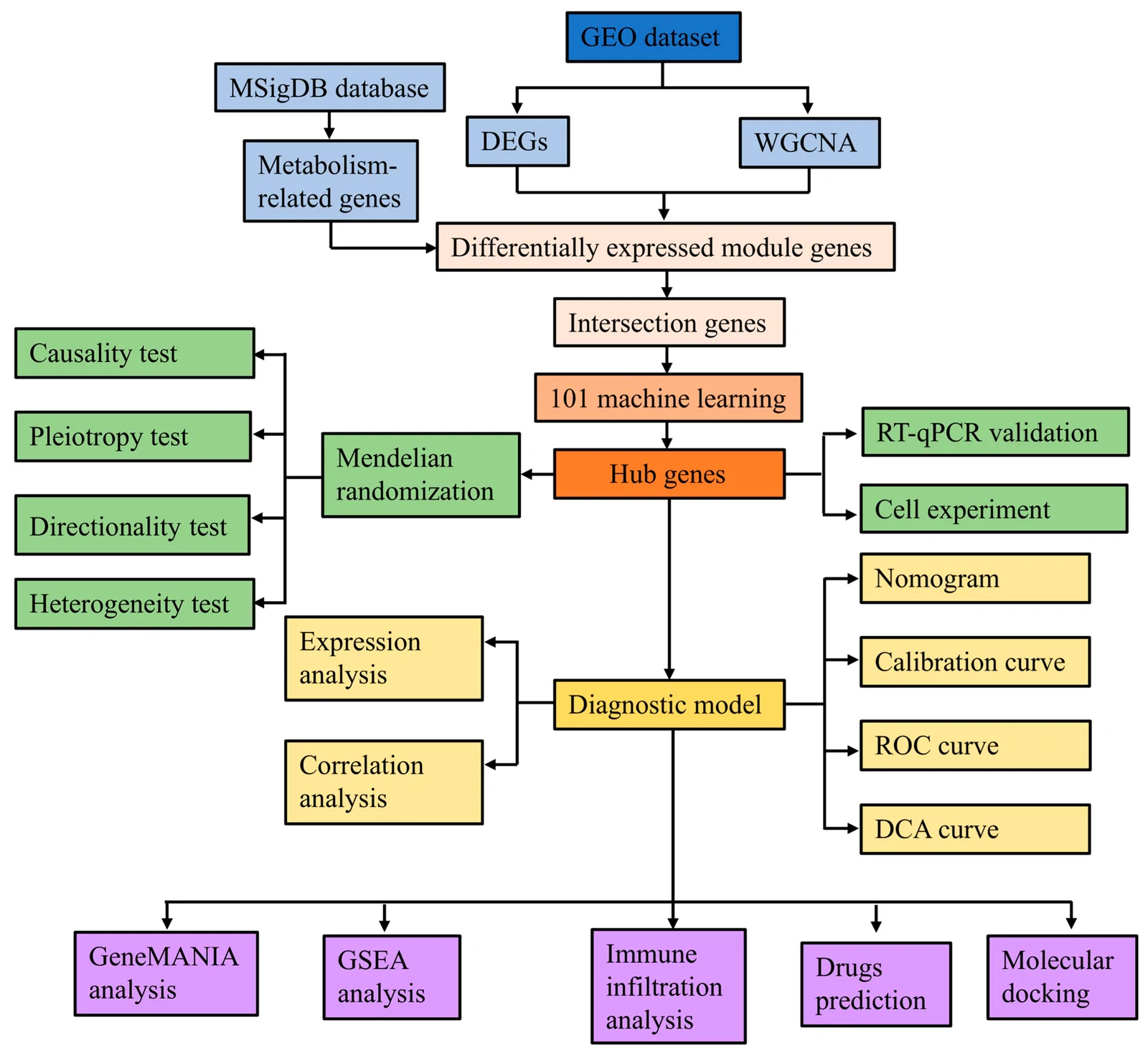
Flowchart of the study design.

**Figure 2:**
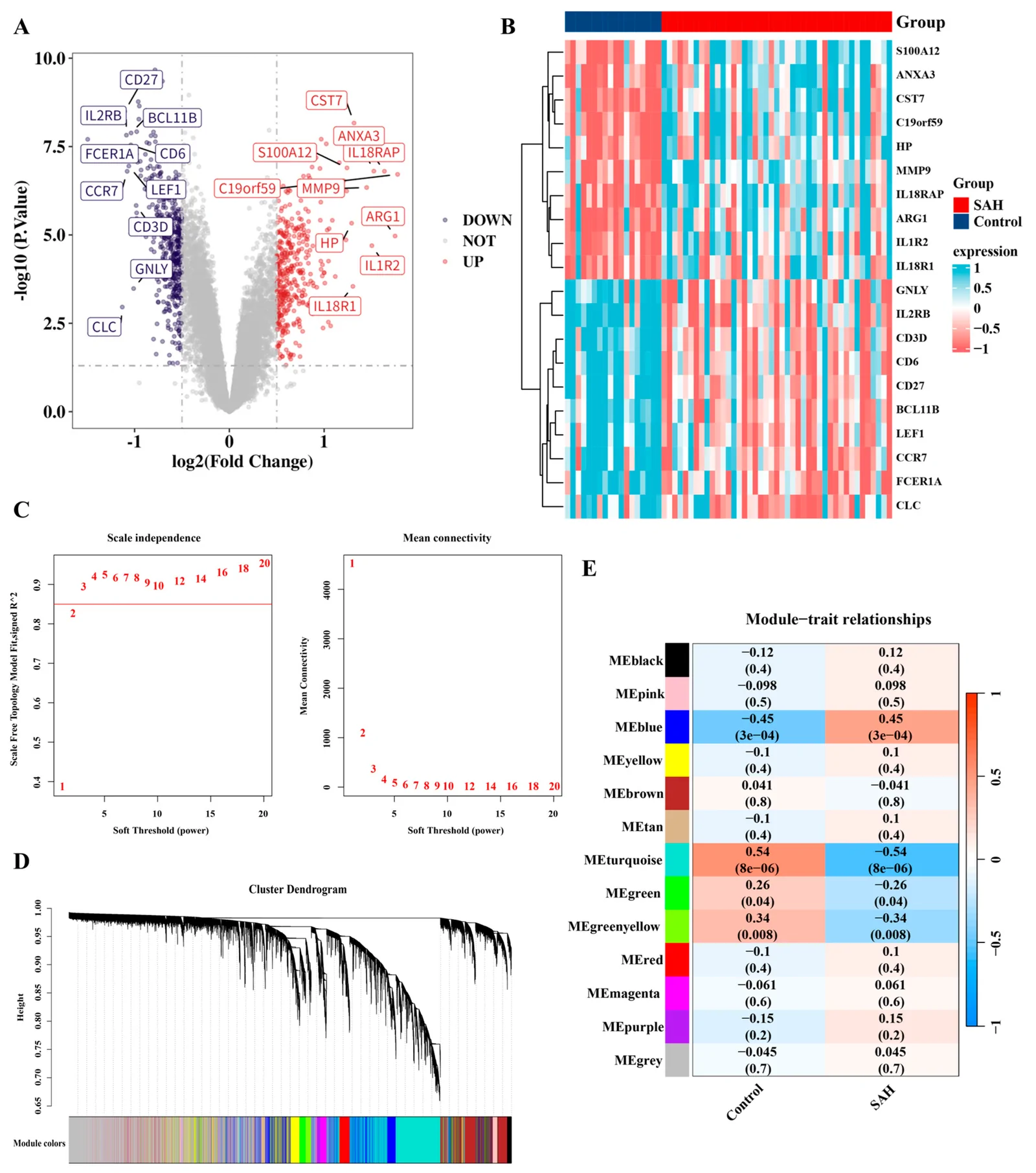
Identification of DEGs and SAH-related module using WGCNA. (**A**) Volcano plot displays DEGs between SAH and control samples. (**B**) Heatmap showing the top 10 DEGs. (**C**) Selection of soft threshold power (β) based on scale independence and mean connectivity. (**D**) Cluster dendrogram of genes, with dynamic tree cutting to identify modules represented by different colors. (**E**) Heatmap showing correlations between gene modules and SAH.

**Figure 3:**
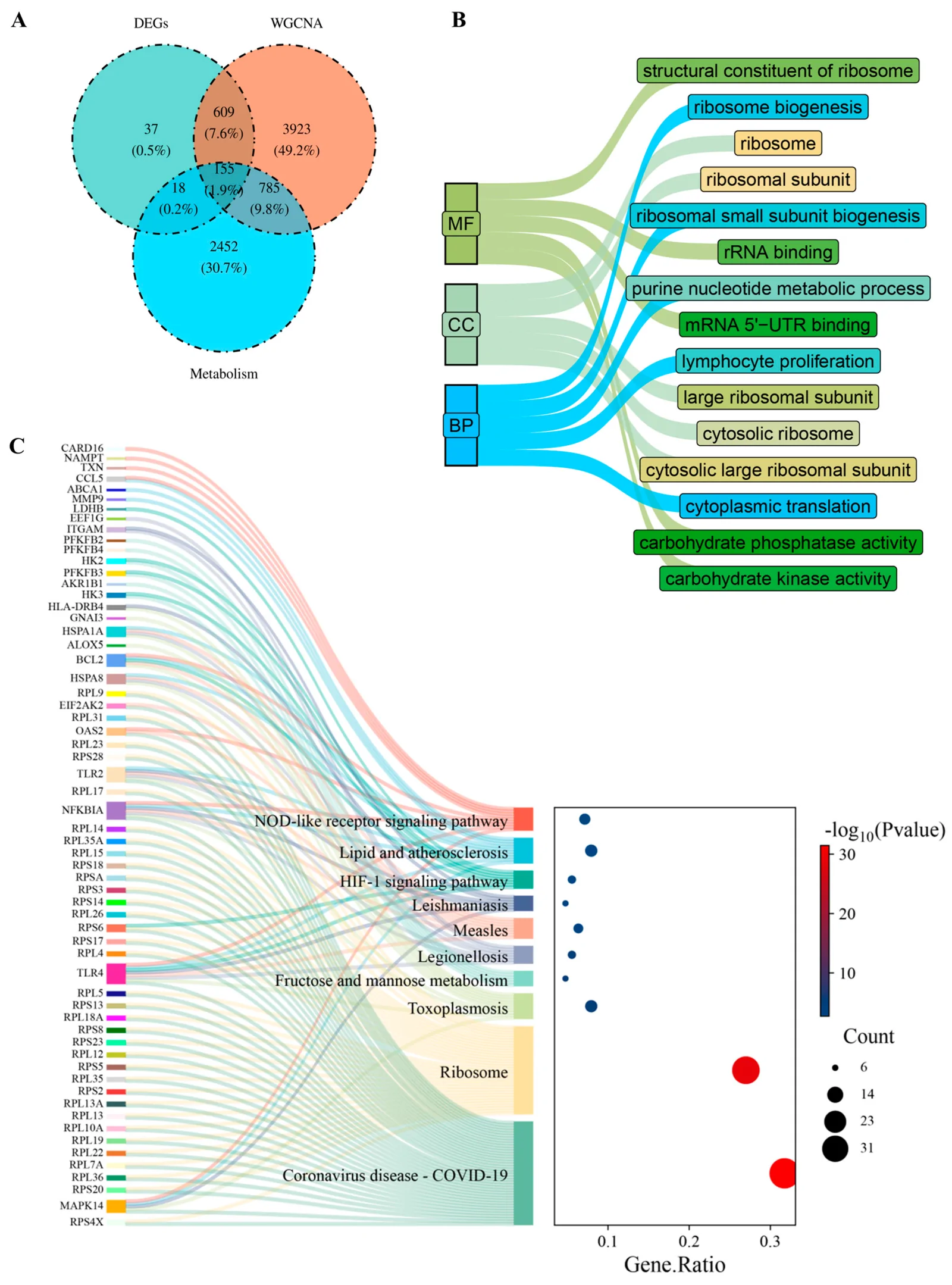

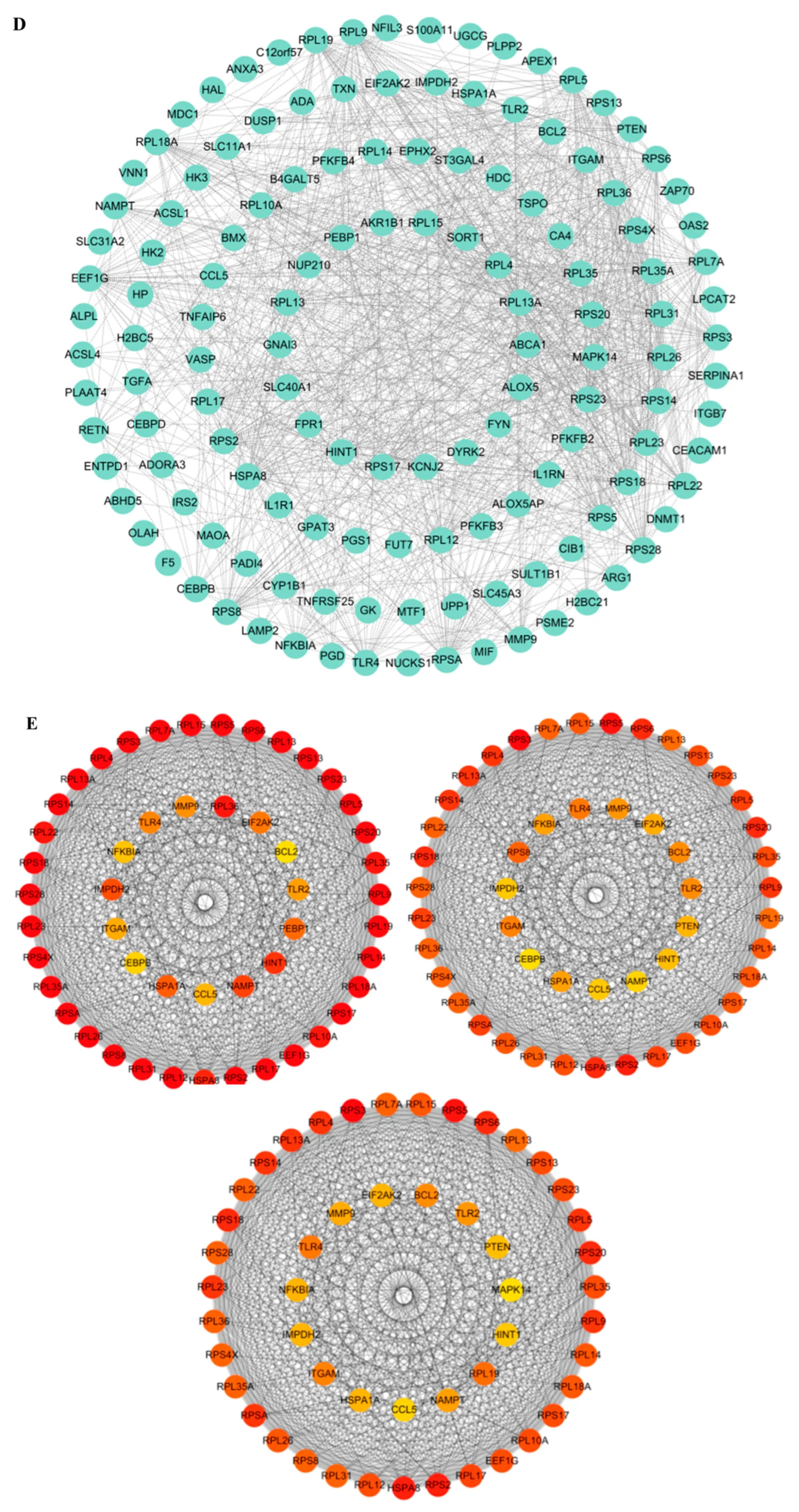
Enrichment and interaction analysis of SAH-related genes. (**A**) Identification of metabolic reprogramming-related DEGs by intersecting DEGs, WGCNA turquoise-module genes, and metabolic reprogramming-related genes. (**B**) GO enrichment analysis. (**C**) KEGG enrichment analysis. (**D**) PPI network diagram of 155 candidate genes. (**E**) Top 50 genes obtained by MCC, Degree, and MNC algorithms. For GO and KEGG enrichment analyses, an adjusted *p*-value (FDR) < 0.05 was considered statistically significant. The transcriptomic data used for DEG and WGCNA analyses were obtained from the GSE36791 dataset (43 SAH patients and 18 controls). All bioinformatics analyses were performed with three technical replicates to ensure reproducibility.

**Figure 4:**
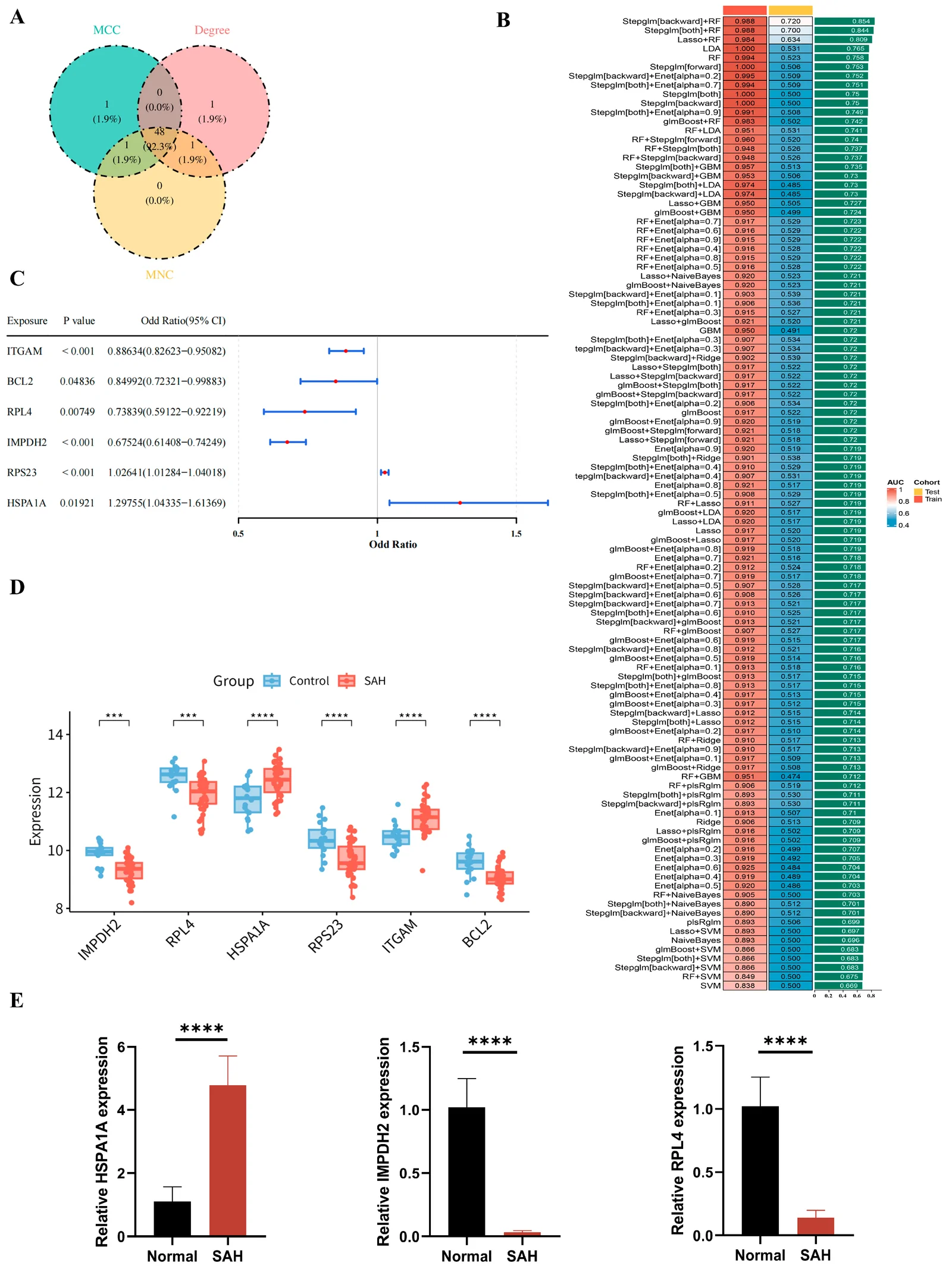
Identification and RT-qPCR validation of three hub genes. (**A**) Intersections of the hub genes obtained by MCC, Degree, and MNC algorithms. (**B**) Screening the optimal model. (**C**) MR analysis results for different exposure variables. (**D**) Expression levels of IMPDH2, RPL4, HSPA1A, RPS23, ITGAM, and BCL2 in the SAH group and control group. (**E**) Expression validation of IMPDH2, RPL4 and HSPA1A by RT-qPCR in clinical samples. RT-qPCR data were analyzed using independent-samples *t*-tests, and data are expressed as mean ± SD. Each group included three independent biological replicates (*n* = 3), and each sample was measured in three technical replicates to assess assay stability. *** *p* < 0.001, **** *p* < 0.0001.

**Figure 5:**
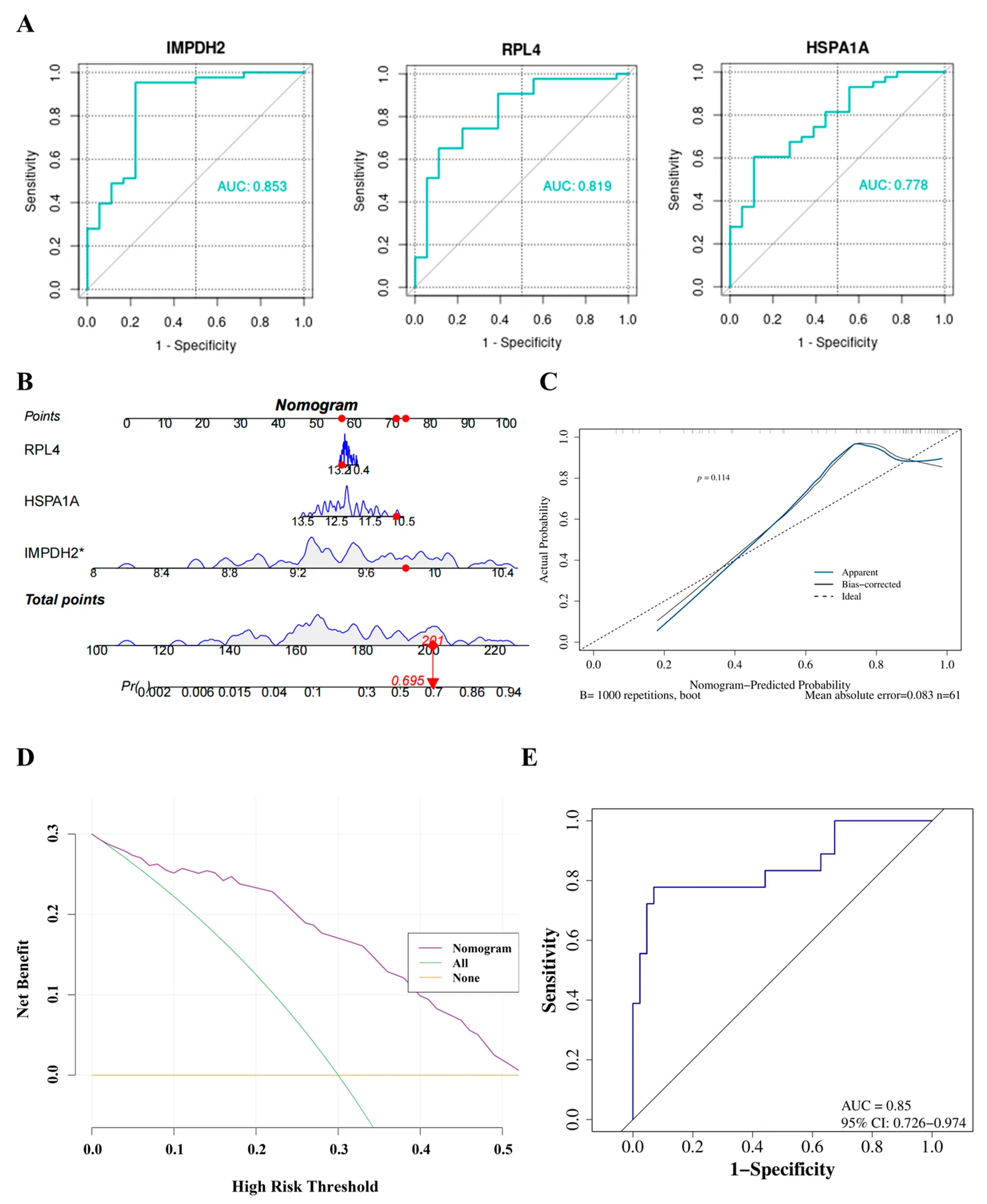
Construction and evaluation of the diagnostic model. (**A**) ROC curves for IMPDH2, RPL4, and HSPA1A, respectively. (**B**) A nomogram model describing the risk of SAH. (**C**) Calibration curve to assess the predictive accuracy of the nomogram. (**D**) DCA curve to evaluate the clinical application value of the nomogram. (**E**) ROC curve for the diagnostic model. * *p* < 0.05.

**Figure 6:**
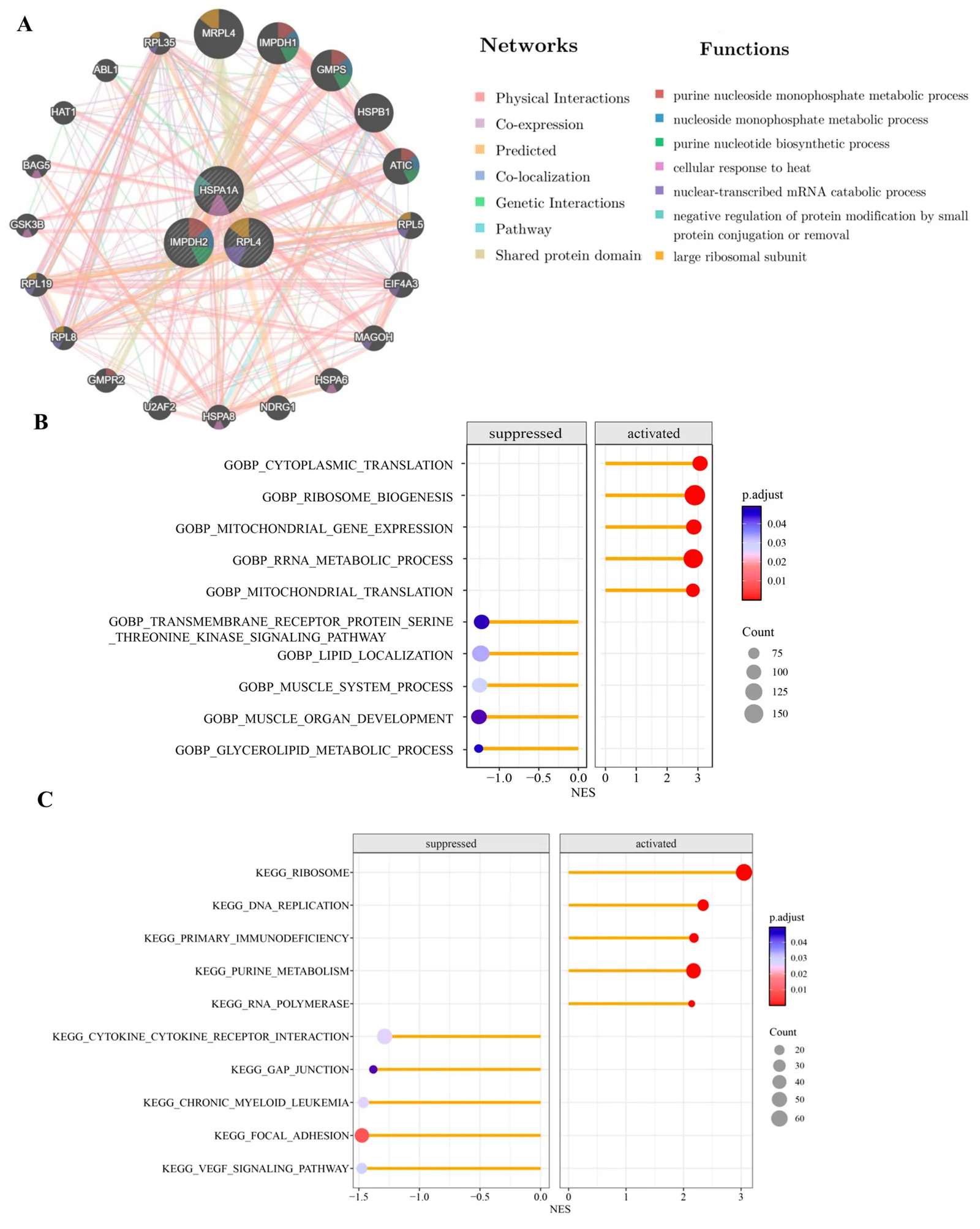

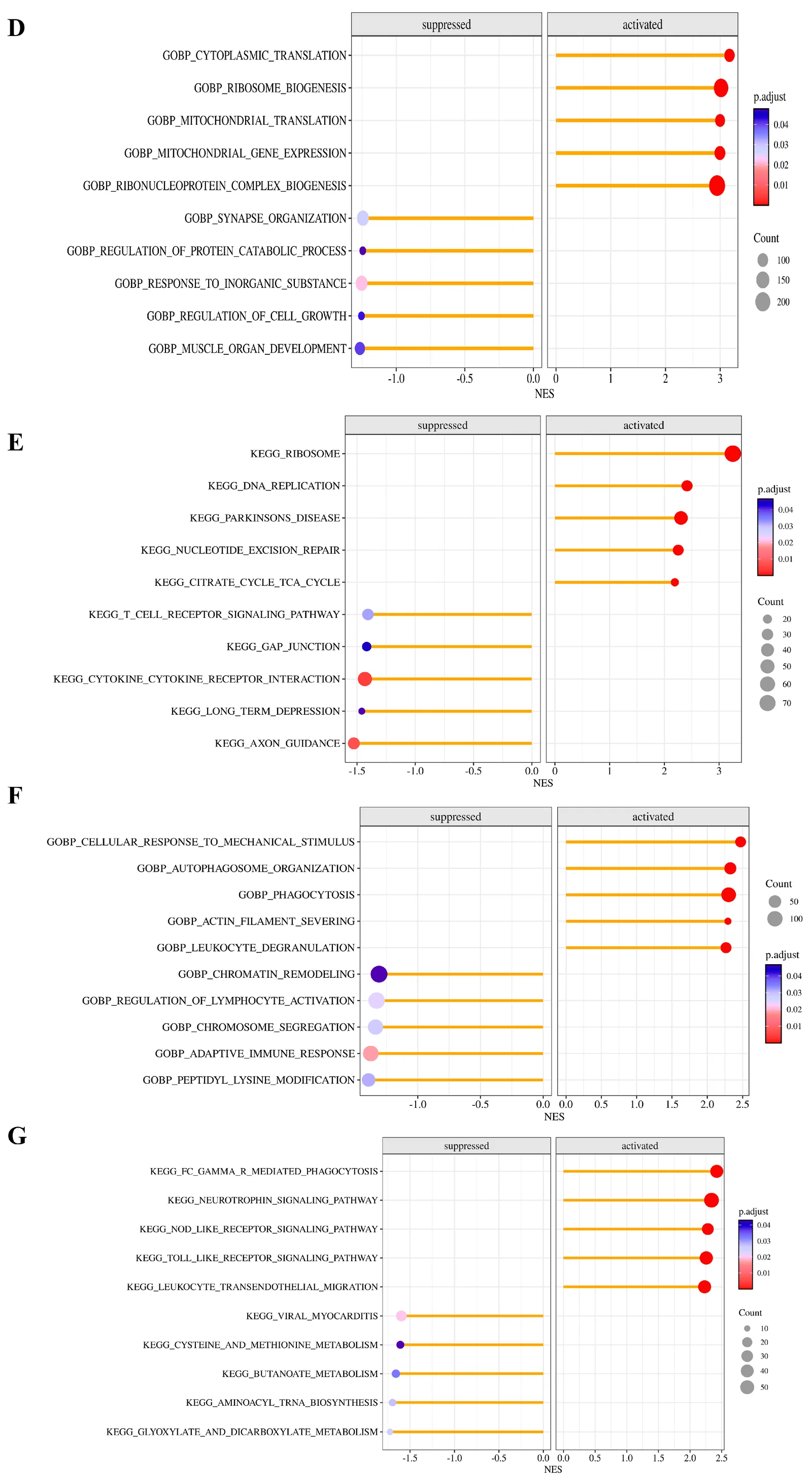
Exploration of biological functions of hub genes. (**A**) Interactions between the three hub genes and related ones, indicating biological pathways and gene relationships associated with SAH. (**B**) GSEA analysis targeting IMPDH2-Go analysis and (**C**) IMPDH2-KEGG analysis. (**D**) GSEA analysis targeting RPL4-Go analysis and (**E**) RPL4-KEGG analysis. (**F**) GSEA analysis targeting HSPA1A-Go analysis and (**G**) HSPA1A-KEGG analysis.

**Figure 7:**
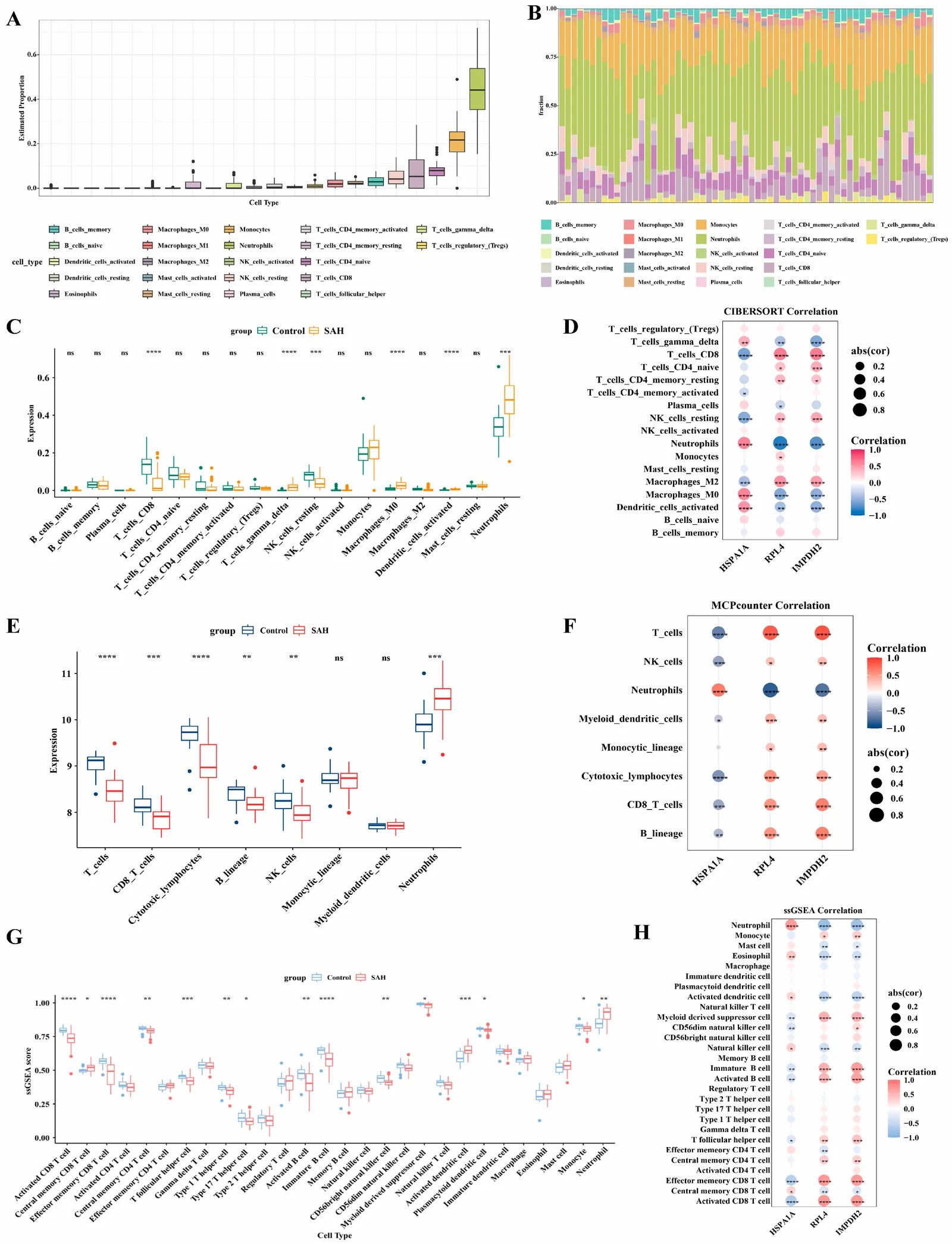
Circulating immune-cell composition analysis and its relationship with the identified hub genes. (**A**) Boxplot of immune cell abundance by the CIBERSORT algorithm; (**B**) Stacked chart of immune cell abundance; (**C**,**E**,**G**) Comparison of immune cell abundance between the SAH group and control group by CIBERSORT (**C**), MCP-counter (**E**), and ssGSEA analyses (**G**); (**D**,**F**,**H**) Heatmap showing the correlation between hub genes and immune cells by CIBERSORT (**D**), MCP-counter (**F**), and ssGSEA analyses (**H**). ns: not significant; * *p* < 0.05, ** *p* < 0.01, *** *p* < 0.001, **** *p* < 0.0001.

**Figure 8:**
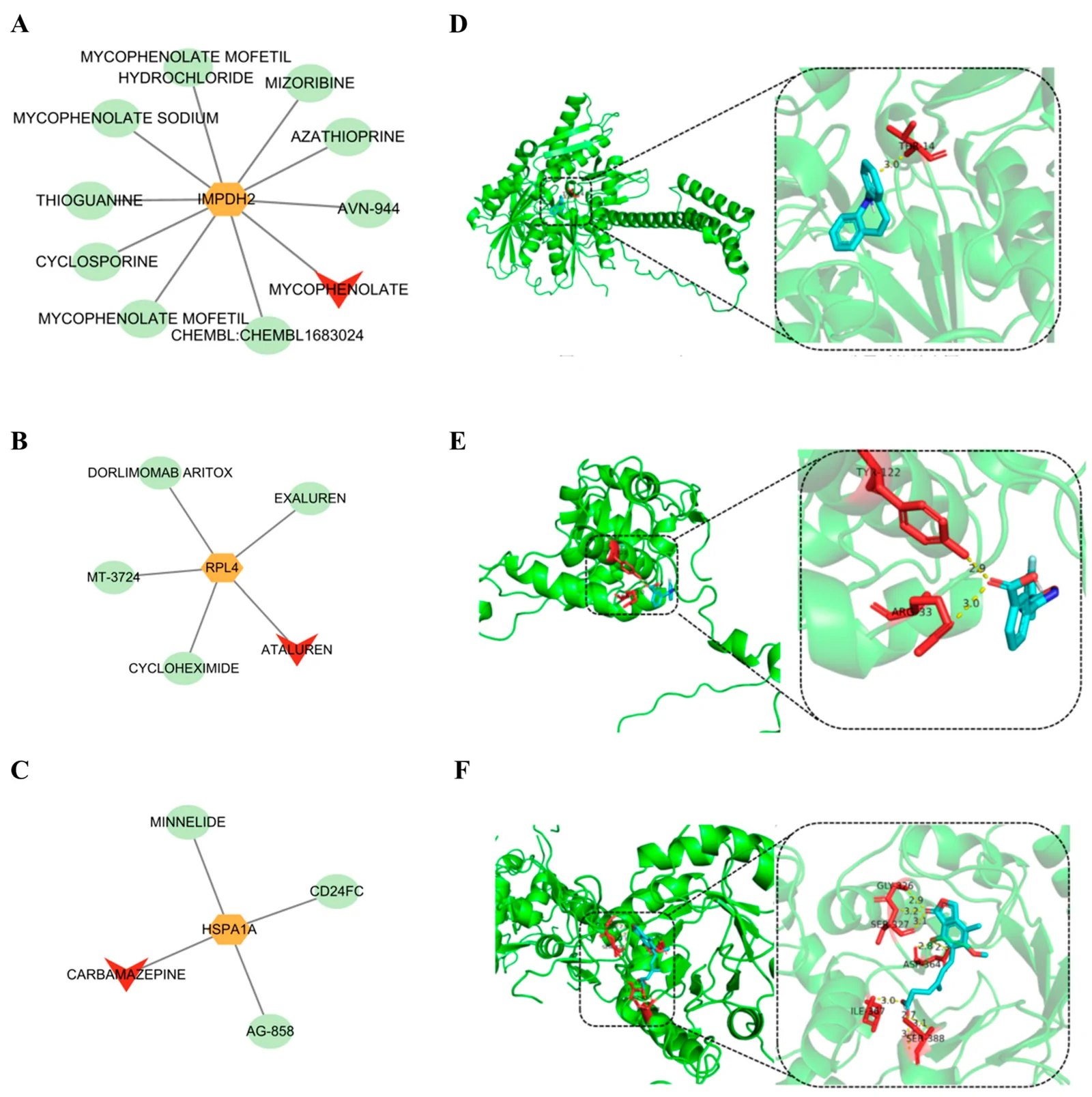
Investigation of potential drugs. (**A**–**C**) Network diagram for hub genes and targeting drugs; (**D**) Molecular docking diagram of carbamazepine binding to HSPA1A; (**E**) Molecular docking diagram of ataluren binding to RPL4; (**F**) Molecular docking diagram of mycophenolate binding to IMPDH2.

**Figure 9:**
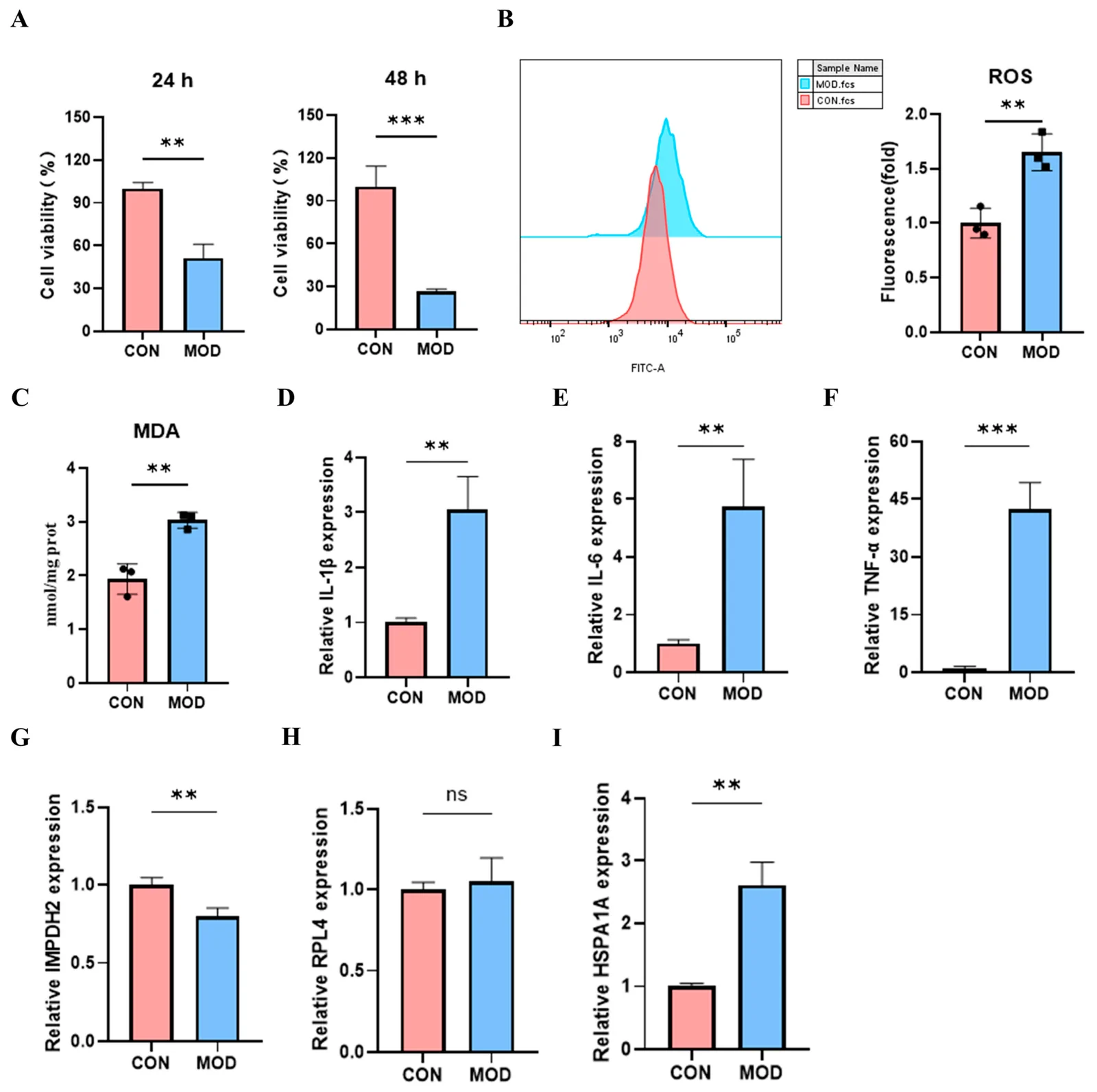
Validation of cellular SAH model and expression levels of hub genes. (**A**) CCK-8 assay for cell viability at 24 h and 48 h; (**B**) Flow cytometry analysis of intracellular ROS levels; (**C**) MDA content measurement. (**D**–**F**) RT-qPCR detection of IL-1β, IL-6, and TNF-α mRNA expression; (**G**–**I**) RT-qPCR detection of IMPDH2, RPL4, and HSPA1A mRNA expression. The data were analyzed using independent-samples *t*-tests for between-group comparisons, and data are expressed as mean ± SD. Each group included three independent biological replicates (*n* = 3), and each sample was measured in three technical replicates to assess assay stability. ns: not significant; ** *p* < 0.01, *** *p* < 0.001.

## Data Availability

The transcriptomic datasets analyzed in this study are publicly available in the NCBI GEO database under accession numbers GSE36791 and GSE37378. The GWAS summary statistics used for MR analysis are available from the IEU OpenGWAS database under accession number ebi-a-GCST90018923. The clinical and experimental data generated in this study are available from the corresponding author upon reasonable request.

## Supplementary Materials

The following supporting information can be downloaded at: https://www.mdpi.com/article/10.3390/genes17101251/s1, Figure S1. GS versus MM for the turquoise module. The scatter plot showed that a strong positive correlation indicates that genes highly connected to the turquoise module are also highly associated with SAH. Figure S2. Scatter plots of MR estimates. Scatter plots showing the associations between genetically predicted expression of candidate genes and the risk of subarachnoid hemorrhage (SAH). Each point represents an instrumental variable, and the horizontal and vertical error bars indicate the standard errors of the SNP effects on gene expression and SAH risk, respectively. The slopes of the fitted lines represent the MR estimates obtained using five methods: inverse variance weighted, MR-Egger, weighted median, simple mode, and weighted mode. Panels show the MR results for ITGAM (A), BCL2 (B), RPL4 (C), IMPDH2 (D), RPS23 (E), and HSPA1A (F). Figure S3. Forest plots of single-SNP MR estimates. Forest plots showing the effects of individual instrumental variables (SNPs) on the associations between genetically predicted expression of candidate genes and subarachnoid hemorrhage (SAH) risk. Black dots indicate the effect estimates for each SNP, and horizontal lines represent the corresponding 95% confidence intervals. The red dot and horizontal line at the bottom of each panel indicate the overall estimate derived from the inverse variance weighted (IVW) method. Panels show the results for ITGAM (A), BCL2 (B), RPL4 (C), IMPDH2 (D), RPS23 (E), and HSPA1A (F). Scatter plot of the correlation between the three hub genes. Figure S4. Leave-one-out sensitivity analysis of MR estimates. Leave-one-out sensitivity analysis showing the associations between genetically predicted expression of candidate genes and subarachnoid hemorrhage (SAH) risk after sequentially excluding each instrumental variable. Black dots indicate the MR estimates obtained after removing each SNP individually, and horizontal lines represent the corresponding 95% confidence intervals. The red dot and horizontal metabolic reprogramming-related line at the bottom of each panel indicate the overall MR estimate using all instrumental variables. Panels show the results for ITGAM (A), BCL2 (B), RPL4 (C), IMPDH2 (D), RPS23 (E), and HSPA1A (F). Figure S5. Correlation analysis among the three hub genes. Scatter plots showing the pairwise correlations among IMPDH2, RPL4, and HSPA1A expression levels in the training cohort. Table S1. Twelve types of metabolic reprogramming-related gene sets. Table S2. Metabolic reprogramming-related genes. Table S3. FDR-corrected DEG list. Table S4. MR estimates and multiple-testing correction. Table S5. The number of instrumental SNPs for each candidate gene. Table S6. The protein structures and chains used for docking. Table S7. The clinical characteristics of participants. Table S8. The sequences of primers. Table S9. Module assignment, MM, and GS for all genes in each module. Table S10. Results of the horizontal pleiotropy test. Table S11. Results of Steiger directionality analysis. Table S12. Results of heterogeneity test.
