# Supplementary material for: Identification and In Vitro Validation of Metabolic Reprogramming-Related Hub Genes for the Diagnosis of Subarachnoid Hemorrhage
Source: Genes (Basel). 2026 Oct 10;17(10):1251. doi: 10.3390/genes17101251 (PMC13704222; doi:10.3390/genes17101251)
Supplement: Supplementary file 1 [file genes-17-01251-s001.zip › Supplementary Table S1, 6, 8, 10, 11 and 12.pdf]

Table S1. Twelve types of metabolic reprogramming-related gene sets

Gene set	Gene number
Amino acid metabolism	374
Cholesterol metabolism	140
Fatty acid biosynthetic process	166
Fatty acid metabolism	158
Lactate metabolism	333
Lipid metabolism	776
Manganese metabolism	1399
Nucleotide metabolism	882
Oxidative phosphorylation	200
Purine metabolism	159
Pyrimidine metabolism	98
Glycolysis	74

Table S6. The protein structures and chains used for docking

Gene	Accession/PDB ID	Protein chain	Ligand	Binding score (kcal/mol)
HSPA1A	AF-P0DMV8-F1	A	CARBAMAZEPINE	-9.4
RPL4	AF-P36578-F1	A	ATALUREN	-7.4
IMPDH2	1B3O	A, B	MYCOPHENOLATE	-7.7

Table S8. The sequences of primers

Primers	Sequences
Human IMPDH2 F	CCGACTACCTGATTAGTGGGG
Human IMPDH2 R	GTCTCCGCAGTTGAAGAGC
Human RPL4 F	ATACGCCATCTGTTCTGCCC
Human RPL4 R	GCTGAGAGGCATAGACCTTTTTG
Human HSPA1A F	AGCTGGAGCAGGTGTGTAAC
Human HSPA1A R	CAGCAATCTTGGAAAGGCCC
Human IL-6 F	ACTCACCTCTTCAGAACGAATTG
Human IL-6 R	CCATCTTTGGAAGGTTCAGGTTG
Human IL-1β F	ATGATGGCTTATTACAGTGGCAA
Human IL-1β R	GTCGGAGATTCGTAGCTGGA
Human TNF-α F	CCTCTCTCTAATCAGCCCTCTG
Human TNF-α R	GAGGACCTGGGAGTAGATGAG
Human β-actin F	CATGTACGTTGCTATCCAGGC
Human β-actin R	CTCCTTAATGTCACGCACGAT

Table S10. Results of horizontal pleiotropy test

NO.	SYMBOL	id.exposure	egger_intercept	se	pval
1	ITGAM	eqtl-a-ENSG00000169896	0.001	0.008	0.885
2	BCL2	eqtl-a-ENSG00000171791	0.024	0.030	0.430
3	RPL4	eqtl-a-ENSG00000174444	0.013	0.042	0.749
4	IMPDH2	eqtl-a-ENSG00000178035	0.030	0.023	0.207
5	RPS23	eqtl-a-ENSG00000186468	0.011	0.006	0.068
6	HSPA1A	eqtl-a-ENSG00000204389	22.501	-0.003	0.941

Table S11. Results of steiger directionality analysis

NO.	SYMBOL	snp_r2.exposure	snp_r2.outcome	steiger_direction	steiger_pval
1	ITGAM	0.215925	0.000142	TRUE	0
2	BCL2	0.069239	0.000227	TRUE	0
3	RPL4	0.021674	2.81E-05	TRUE	5.18E-105
4	IMPDH2	0.110627	0.000185	TRUE	0
5	RPS23	2.664693	0.000334	TRUE	0
6	HSPA1A	0.09789	6.80E-05	TRUE	2.15E-105

Table S12. Results of heterogeneity test

NO.	SYMBOL	id.exposure	method	Q	Q_df	Q_pval
1	ITGAM	eqtl-a-ENSG00000169896	IVW	50.391	77	0.991
2	BCL2	eqtl-a-ENSG00000171791	IVW	85.087	49	0.001
3	RPL4	eqtl-a-ENSG00000174444	IVW	2.130	18	0.999
4	IMPDH2	eqtl-a-ENSG00000178035	IVW	10.870	63	0.999
5	RPS23	eqtl-a-ENSG00000186468	IVW	129.737	191	0.999
6	HSPA1A	eqtl-a-ENSG00000204389	IVW	22.501	31	0.866

IVW, inverse variance weighted.
